# Single molecule targeted sequencing for cancer gene mutation detection

**DOI:** 10.1038/srep26110

**Published:** 2016-05-19

**Authors:** Yan Gao, Liwei Deng, Qin Yan, Yongqian Gao, Zengding Wu, Jinsen Cai, Daorui Ji, Gailing Li, Ping Wu, Huan Jin, Luyang Zhao, Song Liu, Liangjin Ge, Michael W. Deem, Jiankui He

**Affiliations:** 1 Direct Genomics Co., Ltd., Shenzhen, Guangdong, 518055, China; 2 Key Laboratory of Flexible Electronics & Institute of Advanced Materials, Jiangsu National Synergetic Innovation Center for Advanced Materials, Nanjing Tech University, Nanjing, 211816, China; 3 Chemistry Department, North Carolina State University, Raleigh, NC, 27695, USA; 4 Clinical Medical Research Center, the Second Clinical Medical College of Jinan University (Shenzhen People’s Hospital), Shenzhen, Guangdong, 518020, China; 5 Departments of Bioengineering and Physics & Astronomy, Rice University, Houston, TX, 77005, USA; 6 Department of Biology, South University of Science and Technology of China, Shenzhen, Guangdong, 518058, China

## Abstract

With the rapid decline in cost of sequencing, it is now affordable to examine multiple genes in a single disease-targeted clinical test using next generation sequencing. Current targeted sequencing methods require a separate step of targeted capture enrichment during sample preparation before sequencing. Although there are fast sample preparation methods available in market, the library preparation process is still relatively complicated for physicians to use routinely. Here, we introduced an amplification-free Single Molecule Targeted Sequencing (SMTS) technology, which combined targeted capture and sequencing in one step. We demonstrated that this technology can detect low-frequency mutations using artificially synthesized DNA sample. SMTS has several potential advantages, including simple sample preparation thus no biases and errors are introduced by PCR reaction. SMTS has the potential to be an easy and quick sequencing technology for clinical diagnosis such as cancer gene mutation detection, infectious disease detection, inherited condition screening and noninvasive prenatal diagnosis.

In the past few years, the cost of large-scale DNA sequencing has been dramatically driven down by the tremendous advances in next-generation sequencing (NGS)^1^. Nonetheless, the costs of human whole genome sequencing and bioinformatics interpretation are still significant. NGS is very effective for whole genome sequencing and whole exome sequencing. This strategy is not feasible in clinical practice. It is apparently more cost-effective and time-efficient to sequence the specific genomic regions of interest^2^. Several studies have been done recently. For example, there are several commercially available cancer gene panels that target as few as 50 to as many as several hundred genes that frequently mutate in cancer patients^3^. The targeted cancer gene panel sequencing approach proved to be useful in hereditary cancer diagnosis and disease management.

Current NGS based targeted sequencing methods require a separate step for capture enrichment during sample preparation before sequencing^4^,^5^. Enrichment through hybridization and multiplex PCR are two most commonly used customized capture methods. In the solution-based hybridization method, biotinylated DNA or RNA capture probes are designed to combine with the targeted gene fragments, which are then to be purified and collected by streptavidin-labeled magnetic beads. In the multiplex PCR method, the targeted genes could be amplified and collected through PCR reaction which contains the specific primers related to the targeted gene fragments.

In this report, we demonstrated a technology and platform to perform Single Molecule Targeted Sequencing (SMTS), which combined targeted capture and sequencing in one step. A high resolution microscope, Total Internal Reflection Fluorescence (TIRF) microscope, was used to detect the fluorescent probe labelled on the nucleotide in single molecule level. The unwanted background noise was eliminated by TIRF so that single molecule can be observed^6^. Specific primers aiming to capture the genes of interest were attached on the surface of flow cell. Once the DNA targets were hybridized with the primers in flow cell, the sequencing would begin without PCR enrichment which is usually required in traditional sequencing technology. Compared to current targeted sequencing methods with mutiple capture steps, SMTS has the potential advantages of simplified sample preparation and thus it can avoid biases or error introduced by PCR amplification^7^. SMTS technology provides a new platform that is extremely suitable for cancer gene mutation detection, infectious disease detection, inherited condition screening and noninvasive prenatal diagnosis.

## Results

### Single molecule detection

A fundamental limitation to the detection of single molecule fluorescent signals stems from the intrinsic qualities of the fluorophores. The key challenge is to reduce the background interference, which may arise from Raleigh scattering, Raman scattering and contaminant fluorescence. Various fluorescence microscopies have been developed in the last two decades to improve the signal to noise ratio (S/N) in order to detect single molecules in the presence of optical background^8^.

**Figure 1:**
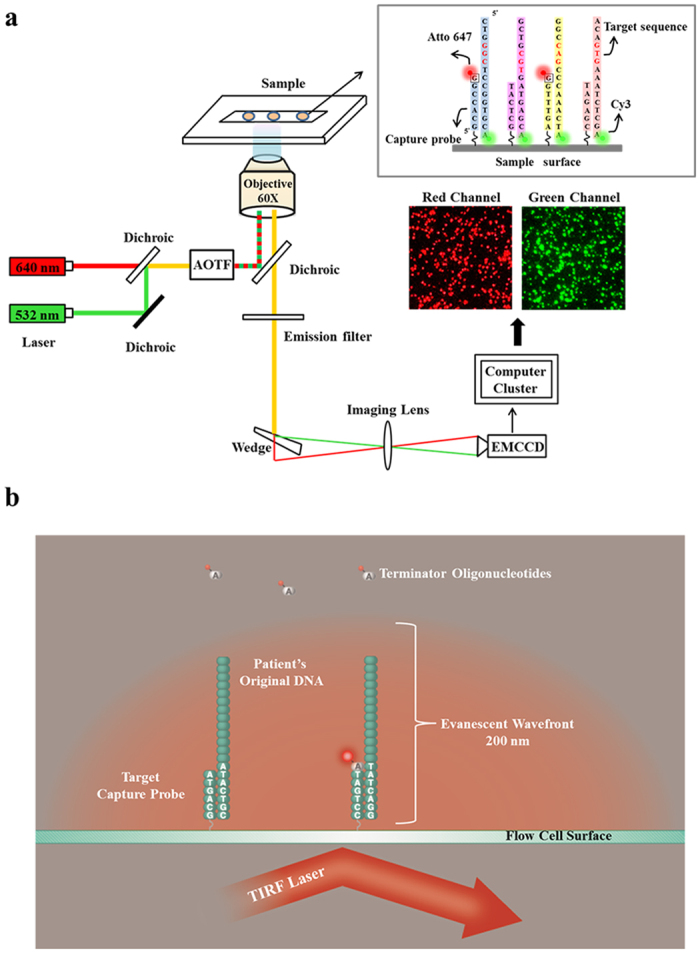
Schematic drawing of single molecule sequencing platform. (**a**) Schematic drawing of the optical setup. The green laser illuminates the Cy3 dyes which are labelled at 3′ end of the targeted DNA template. The Cy3 dyes are non-cleavable. The red laser illuminates the cleavable ATTO647N dyes which are labelled on reversible terminators. Fluorescence images of both Cy3 and ATTO647N are recorded independently by an EMCCD. (**b**) Principle of the single molecule detection by TIRF during sequencing. The capture probes are covalently attached to the coverslip surface, and the target DNA templates are hybridized to the capture probes. The evanescent wave of TIRF only illuminated the area within 200 nm above the flow cell surfaces. Only the DNA templates that are going to be sequenced are within the range of evanescent wave.

We adopted the Total Internal Reflection Fluorescence (TIRF) microscopy in this study. The optical setup is shown in Fig. 1. When light strikes an interface going from coverslip glass to fluid in the flow cell chamber at an incident angle greater than the critical angle, it undergoes total internal reflection. This generates an exponentially decaying light field called the “evanescent wave” above the surface of glass. The evanescent wave excites fluorescent molecules only within ~150–200 nanometers from the surface. The fluorescence from the labelled DNA molecules anchored on the glass surface passes through the filter and the microscope objective and is finally detected by a high sensitive Electron-Multiplying CCD (EMCCD) camera. As only the vicinity of the surface is illuminated, the noise from the bulk fluids in the flow cell chamber is dramatically reduced. Single DNA molecules anchored on the surface can thus be monitored with high S/N ratio(Figs S1–S3).

The choice of fluorescent dyes to label nucleotides is also critical for single molecule detection. Many common fluorescent labels show rather low photostability especially when excitation laser power is high and observation lasts a long period of time. ATTO 647N dye was selected to label the nucleotides, whose fluorescence quantum yield (~0.65)is twice that of Cy5 (~0.32) according to ATTO-TEC. We additionally optimized the imaging buffer to increase the photostability. (Fig. S5).

**Figure 2:**
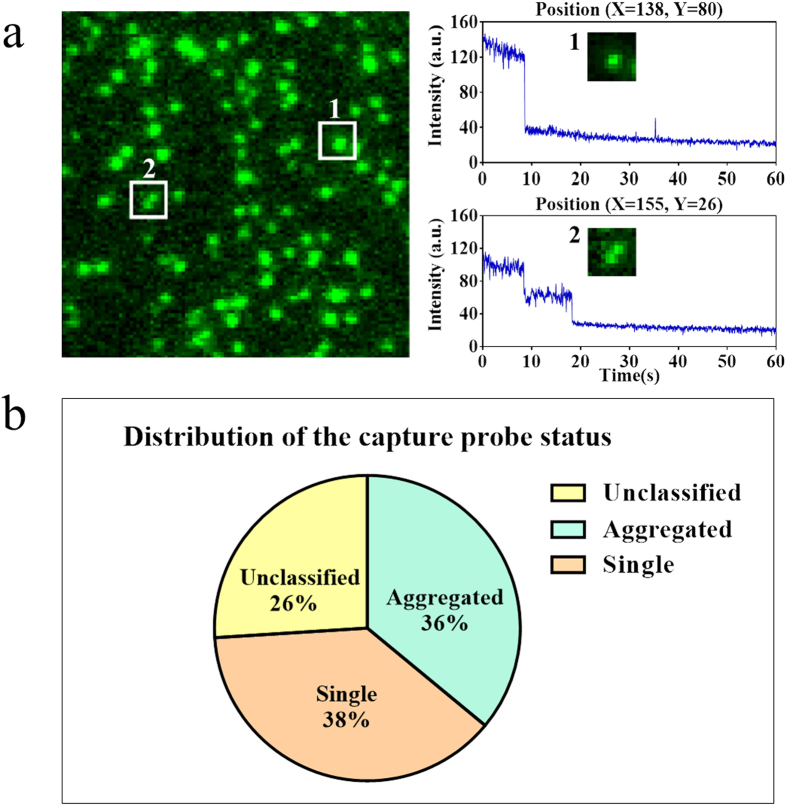
Quantifying the ratio of capture probes that are single molecules. (**a**) The photobleaching of single molecules occurs in a single step. Here, a single spot was traced and its intensity was recorded. A single-step photobleaching indicated that this spot was composed of only one Cy3 molecule, i.e. the spot #1. Spot #2 was composed of two molecules bound together and therefore displayed two steps of photobleaching. (**b**) The composition of single molecules, aggregated molecules and unclassified cases in one field of view.

Single-step photobleaching was used as a quality control to distinguish single molecules from aggregated molecules. In the ideal situation, each DNA molecule was separately bound to the flow cell surface and the minimal distance between the imaged DNA molecule and its neighbours was larger than the diffraction limit of light. In the random chemical attachment procedure used in the present study, two or more DNA molecules may bind to the surface at a distance less than the Rayleigh criterion, simply based on Poisson statistics. We differentiated and quantified the ratio of single DNA molecules to aggregated DNA molecules binding to the flow cell surface by tracing the photobleaching patterns. The basis to differentiate the molecules is that the single molecule photobleaching curve is close to one-step ladder, while aggregated molecules will be photobleached through multiple steps (Fig. 2). We observed that approximate 38% of spots are single molecules. An additional 36% of spots are aggregated molecules. The remaining 26% spots are unclassifiable, due to an irregular photobleaching curve (Figs S1 and S2).

### Targeted hybridization and sequencing

**Figure 3:**
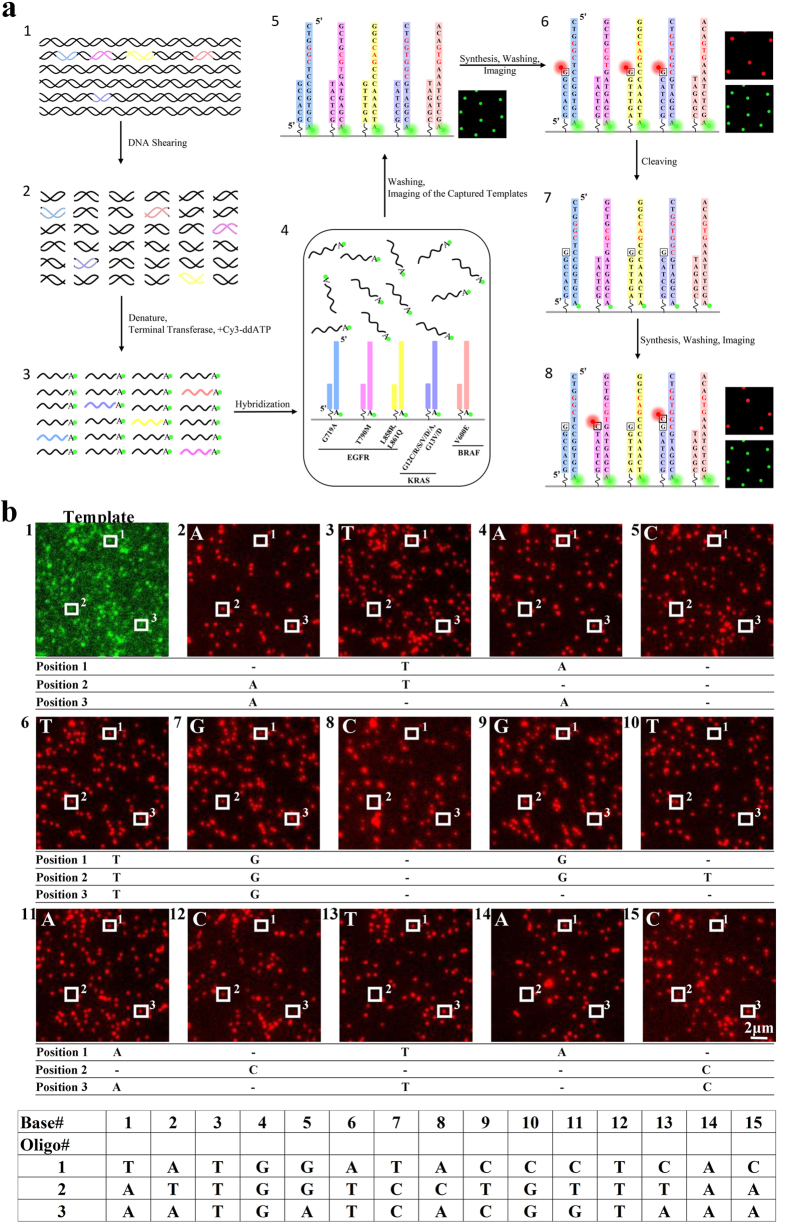
(**a**) The sequencing procedure. DNA template with Cy3 attached at 3′ end was hybridized to the flow cell anchored with capture probes (step 1–4). The capture probes were designed to be complementary to the genes of interest. Unhybridized DNA templates were washed away. The green laser excited the Cy3 fluorescence dye to locate the position of target DNA templates. (step 5). One of the four types of reversible terminators labelled with red fluorescence and polymerases mixture were added to the flow cell. The DNA molecule was extended by a base if the reversible terminator was complementary to the next base in the DNA molecule. Unincorporated reversible terminator was washed away. The red laser excited the ATTO647N fluorescence dyes of reversible terminators (step 6). The fluorescence dyes in the reversible terminators were cleaved and washed away (step 7). A new cycle of sequencing began (step 8). (**b**), Multiple sequencing cycles of base addition, imaging and base calling. We traced a part of one field of view in multiple sequencing cycles. The initial image of Cy3 green fluorescence dyes was used to locate the position of target templates. Three spots were identified and traced as an example. In the first cycle, reversible terminator A (nucleotide analog) was flowed in for reaction. Spot 2 and 3 successfully incorporated a base, indicating that the complementary T was in the same position on the targeted DNA. In the second cycle, the reversible terminator T was flowed in for reaction. Spot 1 and 2 successfully incorporated a base, indicating that the complementary A was in the same position on the targeted DNA. As sequencing continued, the length of DNA template was extended. In this way the sequence of DNA template at spots 1, 2 and 3 was reconstructed.

The EGFR, KRAS and BRAF genes were selected for sequencing in this study. In particular, we aimed to sequence the 8 genetic variants that are related to drug response, including six point mutations and two short deletions (Table 1). Eight capture probe sequences were designed to complement the upstream region of these drug response related mutations. The capture probes were synthesized and anchored to the flow cell surface by chemical bonding. As a principle proof, two sets of targeted DNA templates for sequencing were synthesized. The first set was the wild type sequence and the second set contained mutations and short deletions (Table 1). Each target DNA template contained a Cy3 fluorescence dye at the 3′ end. Excitation of 3′ Cy3 fluorescent dyes was used to mark positions of annealed templates on the flow cell surfaces. Synthetic target DNA templates were hybridized to capture probes which have been attached on the flow cell surface (Fig. 3a).

The sequencing reaction began with locating the target DNA templates, which were randomly hybridized to the surface-bound capture probes (Fig. 3a). The Cy3 fluorescent dyes labelled on the targeted DNA templates were excited by a 532 nm green laser and the images were collected to locate the positions of target DNA templates. Then, modified reversible terminators labelled with ATTO647N and DNA polymerases were added to the flow cell. The reversible terminators were nucleotide analogs containing a cleavable disulfide linker and an inhibitor, which allowed only one reversible terminator to be incorporated into the DNA duplex at one time^9^. Incorporation reaction catalysed by polymerase was carried out at temperature of 37°C, with one of four types of reversible terminators and necessary cofactors. Unincorporated reversible terminators were washed away. The ATTO647N dyes were excited by a 640 nm red laser in an optimized imaging buffer mixture with oxygen scavenger, free radical scavenger, and triplet quenching components^10^,^11^. The images were processed using self-developed software to locate the spot, determine image noise, and filter out false-positive spots. After imaging, the ATTO647N fluorescence dyes were cleaved from the reversible terminators, and the system was ready for a second round of adding another type of reversible terminators and polymerases mixture. The sequencing cycles were repeated until we achieved the desired length of reads (Fig. 3b).

### Sequencing coverage depth

**Figure 4:**
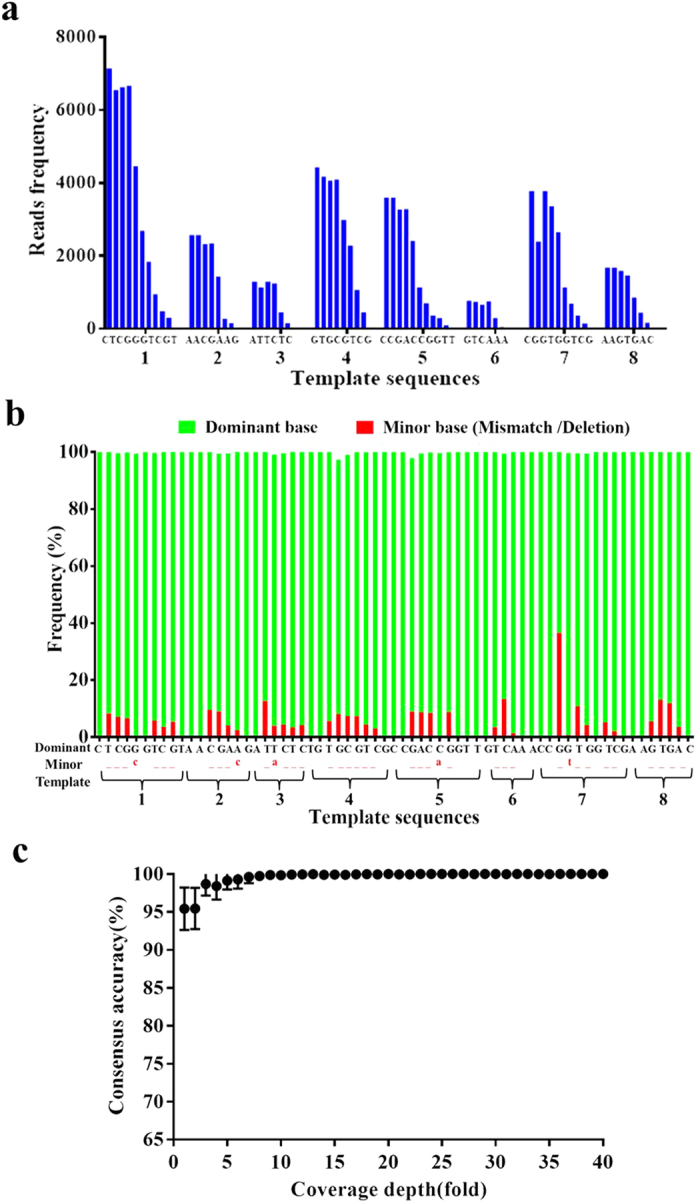
(**a**) The coverage per base. The X-axis is the sequence of 8 templates. The Y-axis is the number of reads at each position. (**b**) The dominant and minor base at each position. The green bars are the dominant bases. The red bars are the minor bases, including only the deletion and substitution bases, represented by underlines and lowercase letters, respectively. (**c**) The consensus accuracy increased with coverage depth. Sampling-subsampling was performed to simulate low coverage situation.

To demonstrate the performance of SMTS, we sequenced the synthesized wild-type EGFR/KRAS/BRAF DNA templates. 19–30 cycles were sequenced, which enabled us to cover all mutation/deletion loci. In each cycle, 300 fields of view (FOV) were imaged. There are approximately 2200–2500 reads in each FOV on average (~0.7–0.8 read/μm^2^). The sequencing reads were aligned to reference DNA sequences with customized program of Smith-Waterman algorithm (Table 2). We observed that the coverage depth varied among the different DNA templates (Fig. 4a). A possible explanation is that the hybridization efficiency for the different DNA templates is sequence-dependent. Additionally the secondary structures formed by the target region can also affect hybridization efficiency. The average coverage depth was 1954-fold. Higher coverage depths could be achieved by capturing images from more FOVs.

### Sequencing accuracy

The accuracy was calculated by comparing the reference sequences with the consensus sequences obtained by SMTS. Consensus sequences were determined by the most frequent base at each position in the sequence alignment (Table 2). By comparing the consensus sequence to the reference sequence base-by-base, the consensus sequence is 100% identical to the reference sequence in each of our four runs when the full sequencing depth was used. We performed sampling-subsampling to the sequence data to get low-coverage data, and recalculated the consensus sequences at different depth of coverage. If each base was covered only once, which means the coverage depth is 1 fold, the accuracy was 95% on average. If each base was covered 5 times or more on average, the consensus accuracy is approached 100% accuracy (Fig. 4c). We performed multiple runs to estimate the type and rates of errors in the raw sequencing data. The reads from each template were separately aligned to the DNA reference sequence. Each position in the reference was mapped by multiple reads. The error rate of a position was defined as the ratio of reads disagreeing with the reference divided by the total number of reads mapped to the reference. The overall error rate was defined as the average of the error rate at all positions. The largest error rates were deletion-type errors (Fig. 4b). The rate of substitution-type errors was relatively small. In four runs, the average substitution error rate is 0.52% per base (Fig. S6).

### Detection of low frequency mutations

**Figure 5:**
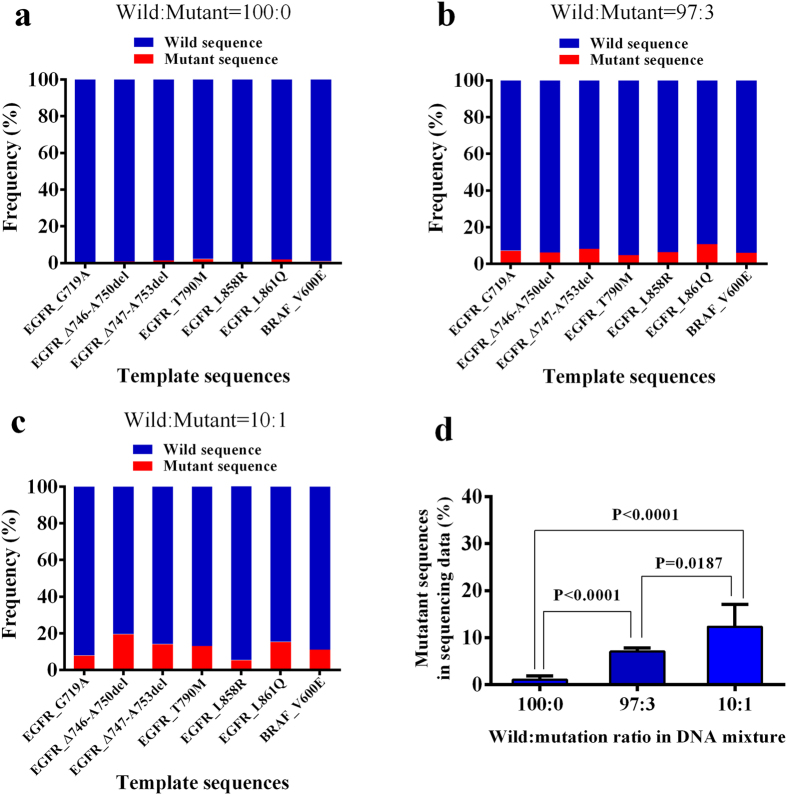
Detecting mutant sequences in a mixture. The wild type and mutant DNA were mixed at 100:0, 10:1 and 97:3 ratios. The mixed DNA was subjected to sequencing. Each sequencing read was aligned to the wild type and mutant type reference sequences and the alignment scores were calculated. If the alignment score of wild type reference sequence was higher than that of mutant type reference sequence, the original sequence read was classified as wild type. Otherwise, it was classified as mutant type. The frequency of wild type and mutant type sequence reads were calculated for each mutant type reference sequence. (**a**–**c**) The frequency of wild type and mutant type sequences calculated from the sequencing data. (**d**) The average of the mutant sequences in the sequencing data over all template sequences.( P value is calculated by two-tailed Student T test based on 0.2 M effective reads).

The wild type DNA was mixed with mutant type DNA at 10:1 and 97:3 ratios (Table 1). The DNA mixture was captured by hybridization in the flow cell and then sequenced. Each raw sequence read was aligned to both wild type and mutant reference sequences to determine its origin. As a control, we also sequenced pure wild type DNA under the same conditions. We found that the percentage of mutant DNA identified in the DNA mixture was significantly higher than that in pure wild type DNA control (Fig. 5). In this experiment, SMTS can detect mutant sequences occurring at a frequency as low as 3%.

## Discussion

Here we demonstrated a method of capturing and sequencing DNA in a single step, which provides a simple approach to targeted sequencing. Results show that the mutations and short deletions model can be successfully detected at frequencies as low as 3%.

Several mutant versions of EGFR/KRAS/BRAF genes were tested in this study. These mutations are actionable and are considered as therapeutically targets. Somatic mutations in EGFR in exon 18, exon 19, exon 21 and the T790M point mutation in exon 20 are predictive of a clinical response to the EGFR tyrosine kinase inhibitor drugs gefitinib and erlotinib^12^,^13^. Somatic mutations in KRAS (codons 12, 13) and BRAF (V600E) in colorectal cancer predict poor prognosis and nonresponse to anti-EGFR antibodies. BRAF V600E is predictive of a positive response to the BRAF V600-specific inhibitor vemurafenib in melanoma^14^.

SMTS has several potential advantages over the traditional Sanger sequencing and other NGS platforms commonly used for the detection of mutations. Firstly, the sample preparation procedure is simple and quick. Only three simple steps are needed. First, the genomic DNA is sheared by sonication. Second, the DNA fragment is denatured to produce single-strand DNA fragment. Third, a ddNTP labelled with Cy3 is ligated to the denatured DNA fragment. In the case of nucleic acids from sources such as FFPE or cfDNA, it is possible that even the sonication step is not necessary. Other high throughput sequencing technologies require one to multiple days of sample preparation, with multiple steps such as sonication, end repairing, dA tailing, adaptor ligation, PCR amplification and target enrichment. Therefore, the SMTS technology has the potential to reduce cost, turn-around time and the risk of errors in sample handling. Moreover, SMTS technology directly sequences the original sample molecules, not PCR amplified products. This direct approach should provide increased sensitivity for the detection of low prevalence mutations and avoid PCR biases^15^, both of which are essential features in the sequencing of a heterogeneous cancer sample^16^. Recently, Oxford Nanopore company released their Beta testing version of sequencer, which may reach a sequence length of over 100,000 base pairs. Compared to other single molecule sequencing technologies such as Oxford Nanopore or Pacific Biosciences, the SMTS is unique in directly capturing and sequencing the genes of interest in one flow cell. The SMTS has less sequencing errors compared to the Oxford Nanopore and Pacific Biosciences sequencer.

We observed that the coverage depth was not uniform among the different positions. Some sequences appeared to show higher error rates than others. The uniformity of coverage could be improved by carefully designing the capture probes, in particularly, to avoid the problematic secondary structure. We also observed that only one third of fluorescence spots were from single molecules. Under the random attachment scenario described in this study, a large portion of the spots came from two or more molecules binding closer than the diffraction limited resolution of the system. The ratio of single molecules could be increased by optimizing the hybridization conditions or controlling the density of capture probes. The read length of SMTS is around 10 bp, since fewer than 30 cycles were performed. This limited number of cycles was performed because the capture probes were designed close to the mutation site of the EGFR/KRAS/BRAF genes, all the mutation sites could be detected within 10 bp. Longer read lengths could be achieved by additional cycles of sequencing. The overall error rate of raw sequences was still significant^17^. To reduce the error rate, the biochemical reaction conditions could be optimized for incorporation of the reversible terminators and cleavage of the fluorescence dye after imaging. Meanwhile, by computational modelling of the error profile, a better base calling algorithm could be developed. The four reversible terminators (A, T, C and G) used in current study were labelled with the same fluorescence dye. In future, we can modify the reversible terminators so that each of the four nucleotides has an unique fluorescence dye^18^. The speed and accuracy could be improved distinctly once the above optimization could be achieved.

Detection of the low frequency mutations has significant value in clinical diagnosis. Traditional Sanger sequencing can reliably detect mutations only when they are present at frequencies greater than ~20%. However, this will not be the case, for example, for a heterozygous mutation in a tumor contaminated with 60% normal DNA, a scenario that is not uncommon. The digital PCR is the most sensitive method to detect point mutation, which can achieve a detection limit of 0.1% or even better^19^. However, the digital PCR can only detect a limited number of mutation sites simultaneously, which restrict its application. Other NGS technology such as Illumina sequence platform can reach a detection limit 1–5%, which is close to the results we got in this study. The ability to detect low frequency mutations will help physicians to choose better strategies for treatment. However, the detected mutation ratios are all significantly higher than the real values. The possible reason might be the following. First, sequencing error will lead to the confusion of wild and mutant type. Second, the algorithm of sequence alignment for single molecule sequencing need to be optimized because the majority of open-source sequence alignment software are designed for Next Generation Sequencer, which has different error profile from single molecule sequencing.

For the foreseeable future, the high cost and complexity of data analysis will limit the application of whole-genome sequencing for the detection of mutations in clinical tests. Targeted sequencing of the regions of interest will therefore remain a key to both detection and prognosis. SMTS is a stride forward in putting this personalized genomic medicine in practice. Although currently only a few loci of specific genes are screened, there is clearly a scope for creation of multi-gene capture arrays, allowing the large number of loci to be analyzed rapidly and cost-effectively with low DNA input requirements. A single step protocol for capturing and sequencing of the whole exome is also possible in future.

## Methods

### Optical setup

A custom-engineered sequencer prototype was developed. It contained a Total Internal Reflection Fluorescence (TIRF) microscope with 60× oil objective (Nikon Ti-E, Japan), EMCCD camera with a resolution of 512 × 512 (Andor, Belfasst, UK) and a 2 color laser powers with 532 nm (100 mW) and 640 nm (100 mW) illumination. A motorized stage (ASI, Eugene, OR, USA) was installed on the TIRF microscope to hold and control the motion of the flow cell (Bioptechs, Bulter, PA, USA) during sequencing. The heater (Bioptechs, Bulter, PA, USA) for both flow cell and objective was installed to maintain the temperature in chamber of the flow cell at 37 °C.

### Flow cell and liquid handing

The FCS2 flow cell contained the chemical functionalized coverslip with epoxy layer (Schott, Jena, Germany), 0.175 mm thick and 40 mm in diameter. A gasket was placed between the coverslip and an aqueduct slide which forms the chamber (3 mm × 23 mm × 0.25 mm) for sequencing reaction. The sandwiched structure was fixed by a top with stainless steel tube inside (inlet port and outlet port) and metal base. A Titan EZ valve with 12 channels (IDEX Health & Science, Oak Harbor, WA, USA) connected the sequencing reagents to the inlet of the flow cell. The outlet of the flow cell was connected with a syringe pump (Tecan, Männedorf, Swiss) to drive the fluidic system by suction.

### Surface chemistry

Synthesized capture probes (oligonucleotides) were covalently bonded to the epoxy coated coverslip surface. The capture probes were firstly incubated at 95°C, then the coverslip was immersed into the capture probe solution with the concentration of 1 nM in K_2_HPO4 buffer (150 mM, pH = 8.5) at 37°C for 2 hours. Then the coverslip was rinsed with 3× SSC, 0.1% Triton X-100 and 3× SSC and 150 mM K_2_HPO4 at pH = 8.5 in sequence.

### Imaging processing

Images were processed using a home-written spot localization algorithm (Fig. S4). Firstly, stage drifts between different imaging cycles were corrected by calculating the peak position of two images by Phase-Only Correlation (POC) function. After aligning all cycles with the first cycle, the corrected images were convolved with a Gaussian kernel. The images were then subjected to a threshold determined by a noise measurement on those images. All contiguous groups of pixels above the threshold were grouped as spots. After that, each spot was fitted with a Gaussian function. This step allowed an accurate determination of the centroid position for single molecules or closely standing molecule pairs. At the same time, clusters of three or more molecules were filtered out. A spot that appeared twice at a same time point but under different wavelength lasers was considered as a base incorporation event. Thus, the spot was renamed as an incorporation spot and marked on the incorporation image. A set of incorporation spot centroids falling within a 1.6 pixel radius in subsequent images is called a “track”. These “tracks” were converted to the final sequences by comparing to the order of adding reversible terminators.

### Target template of EGFR/KRAS/BRAF

Eight mutation sites in three genes(EGFR, KRAS and BRAF)were covered in the designed target templates, including six point mutations(G719A in EGFR exon 18, T790M in EGFR exon 20, L858R and L861Q in EGFR exon 21, G12S and G13D in KRAS exon 2 and V600E in BRAF exon 15) and two short deletions(ΔE746-A750 deletions and ΔE747-A753 deletions in EGFR exon 19). We designed both wild type and mutant target sequence for each genetic variant. The length of each target template was 70 nt with a Cy3 fluorescence dye labelled at the 3′ end. Synthetic target templates were hybridized with the capture probes attached on the surface of flow cell.

### Capture probe design

The capture probes were designed to be complementary to the target sequences. In particular, a 60 nt capture probe sequence including dT10 oligonucleotides and an amine at 5′ end was designed to be complementary to the upstream gene sequence of mutation sites. The 50 nt specific captures’ sequence was designed with the program BatchPrimer3, keeping GC content within 20–80% and Tm’s >65 °C. Capture probes and target templates were synthesized by Sangon Biotech (Shanghai, China).

### Reversible terminators

The modified reversible terminators are composed of nucleotide triphosphates, modified with a detectable label (ATTO647N) by disulfide linker and an inhibitor group. The inhibitor region has multiple negative charged groups (carboxyl group) allowing incorporation of one nucleotide into the DNA duplex while prohibiting the second, third or even more nucleotide incorporation. The detectable label and inhibitor group were cleavable.

### Sequencing cycle

The synthesized templates labelled with Cy3 with a concentration of 5 nM in 3× SSC, pH = 7.0 were introduced into the flow cell and incubated at 55 °C for 2 hours to form a DNA duplex. Then the flow cell was washed with rinse buffer 1, followed by rinse buffer 2.

The sequencing process was controlled automatically including the manipulation of the fluidic system and imaging process Nine pre-prepared reagents were used and stored at two different temperatures. Seven of them are the chemical or biochemical reaction reagents, including four nucleotides (dNTP-ATTO647N) and DNA polymerase mixtures, cleavage reagent (TCEP, 50 mM), cap reagent (50 mM iodoacetamide), and imaging buffer (50 mM Trolox, 15 mM DABCO, 50 mM NaI, 20 mM glucose and 5 mM glucose oxidase in HEPES buffer) stored at 4°C. The other two are rinse buffers including rinse buffer 1 (150 mM HEPES, 1× SSC and 0.1% SDS, pH = 7.0) and rinse buffer 2 (150 mM HEPES and 150 mM NaCl, pH = 7.0) stored at room temperature^20^.

To start the sequencing process, 0.25 μM reversible terminators (one of G, C, T and A) and 20 nM Klenow fragment Exo-minus polymerase(New England Biolabs) mixture was introduced into the flow cell, incubated for 4 minutes at 37 °C and washed out by rinse buffer 1 and 2. Then imaging buffer was pumped in the flow cell and images of 300 fields of view (FOVs) were acquired. Typically, 4 images were taken with 0.1 second exposure time at each FOV (54.6 μm × 54.6 μm). After imaging, the flow cell was washed with rinse buffer. Cleave reagent was introduced into the flow cell and allowed to react for 5 minutes, followed by the capping reagent that was allowed to react for another 5 minutes. Finally the flow cell was washed with rinse buffer again. This completed the first cycle of sequencing. This sequencing cycle was repeated with different reversible terminators. In this paper, the terminators were added into the system in the order of G, C, T, A.

### Bioinformatics

Quality control of the sequence reads was performed. First, reads with length less than 5 bases were filtered out. Second, sequencing reads that appeared fewer than 4 times were filtered out. Third, sequencing reads that could not be aligned to reference sequences were not included for further analysis.

The alignment described above was performed with the Smith-Waterman algorithm, which performs local sequence alignment. By using a customized scoring system (which included a substitution matrix and the gap-penalty scheme), the algorithm successfully achieved optimal local alignment. In this setup, the penalties were −1 for a deletion in a read, −1 for an insertion, 2 for a match and −2 for a substitution. The sequence that had the highest score when aligned to the target template sequences was considered as the called sequence.

## Additional Information

**How to cite this article**: Gao, Y. *et al*. Single molecule targeted sequencing for cancer gene mutation detection. *Sci. Rep.* **6**, 26110; doi: 10.1038/srep26110 (2016).

## Supplementary Material

Supplementary Information

## Figures and Tables

**Table 1:**
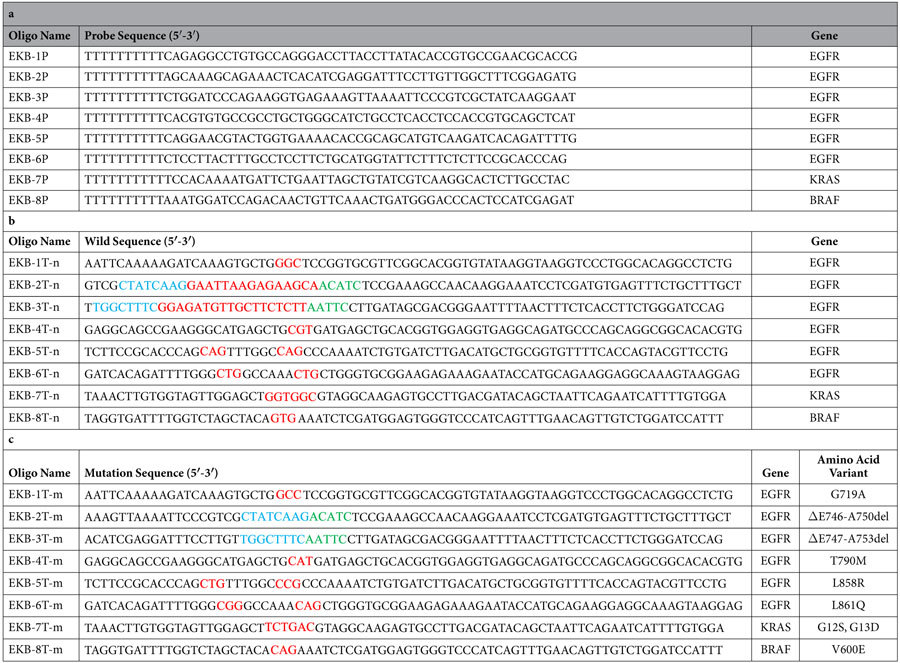
The capture probe and target DNA sequence information.

(a), The synthesized capture probe sequences. The capture probes were designed to capture EGFR/KRAS/BRAF genes. The target DNA sequence designed for testing.

(b), Sequences designed based on the wild type of EGFR/KRAS/BRAF genes. Nucleotide bases in red color are drug-related mutation sites.

(c), Target DNA sequence designed based on the mutant type.

**Table 2:**
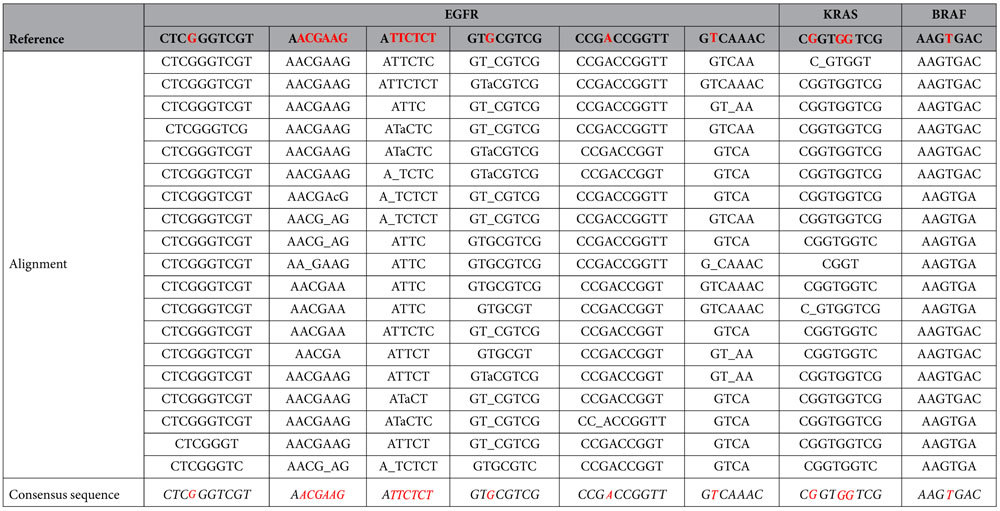
Sequencing alignment of raw reads.

The top row is the reference sequence. Insertion errors are not shown in the alignment.
