# Supplementary material for: Single molecule targeted sequencing for cancer gene mutation detection
Source: Sci Rep. 2016 May 19;6:26110. doi: 10.1038/srep26110 (PMC4872154; doi:10.1038/srep26110)
Supplement: Supplementary Information [file srep26110-s1.pdf]

Supplementary Materials

Single molecule targeted sequencing for cancer gene mutation detection

Yan Gao[1], Liwei Deng[1], Qin Yan[1], Yongqian Gao[2], Zengding Wu[1], Jinsen Cai[1], Daorui Ji[1], Gailing Li[1], Ping Wu[1], Huan Jin[1], Luyang Zhao[3], Song Liu[4], Liangjin Ge[1], Michael W. Deem[5], Jiankui He[6,1],*

[1]Direct Genomics Co., Ltd., Shenzhen, Guangdong, 518055, China
[2]Key Laboratory of Flexible Electronics & Institute of Advanced Materials . Jiangsu National Synergetic Innovation Center for Advanced Materials, Nanjing Tech University, Nanjing, 211816, China
[3]Chemistry Department, North Carolina State University, Raleigh, NC, 27695, USA
[4]Clinical Medical Research Center, the Second Clinical Medical College of Jinan University (Shenzhen People's Hospital), Shenzhen, Guangdong, 518020, China
[5]Departments of Bioengineering and Physics & Astronomy, Rice University, Houston, TX, 77005, USA
[6]Department of Biology, South University of Science and Technology of China, Shenzhen, Guangdong, 518058, China
corresponding. hejk@sustc.edu.cn

Signal-to-Noise ratio

To experimentally test the Signal-to-Noise(S/N) ratio for Cy3 and ATTO647N fluorescence dyes, we performed a photobleaching experiment. The exposure time we used is 100 ms. Sampling frequency is 10MHz. In Fig. S1 and S2, 20 spots of both Cy3 and ATTO647N were randomly selected from the same field-of-view (FOV), with a size of 54.6 µm X 54.6 µm. The S/N ratio for each spot was defined as the average of fluorescence intensity before quenching over its standard deviation.

Results were shown in Fig. S1-S3. Fig. S1 shows 22 spots traces for Cy3. Fig. S2 show 22 spots traces for ATTO647N. Fig. S3 shows the average S/N ratio and standard error for both Cy3 and ATTO647N. As shown in Fig. S3, the average S/N ratio for Cy3 is 5.30, standard error is 0.36. The average S/N ratio for ATTO647N is 13.52, and the standard error is 0.85.

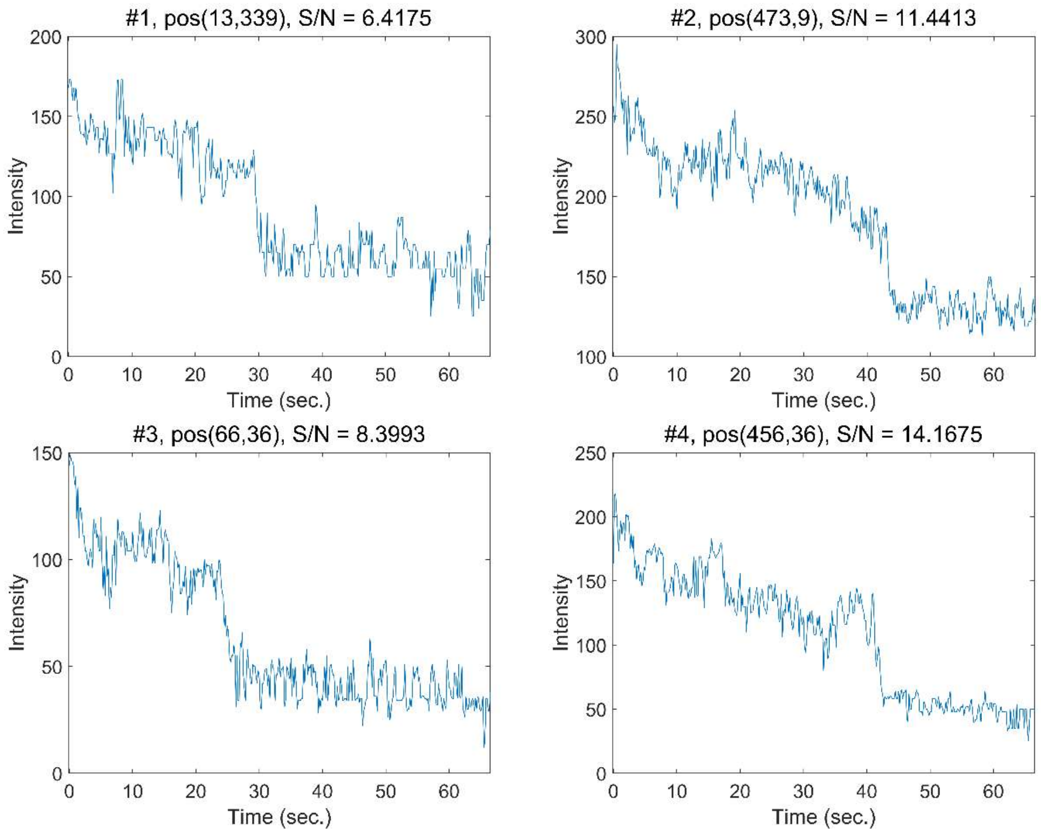

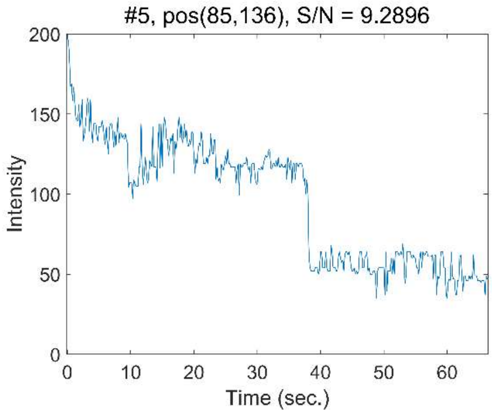
#5, pos(85,136), S/N = 9.2896
Intensity
Time (sec.)

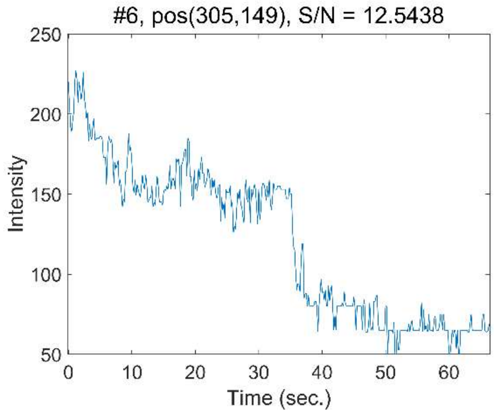
#6, pos(305,149), S/N = 12.5438
Intensity
Time (sec.)

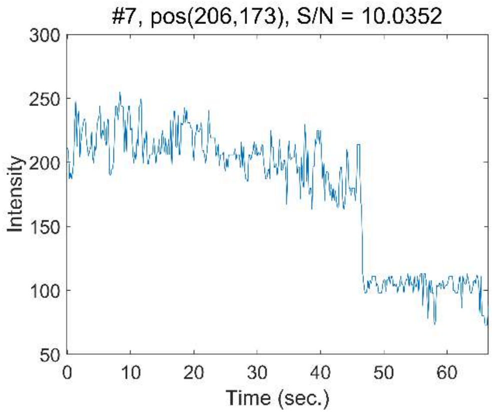
#7, pos(206,173), S/N = 10.0352
Intensity
Time (sec.)

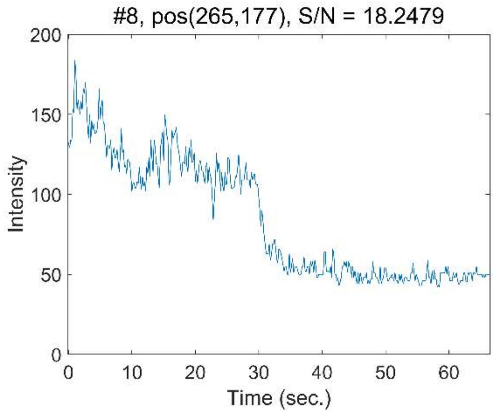
#8, pos(265,177), S/N = 18.2479
Intensity
Time (sec.)

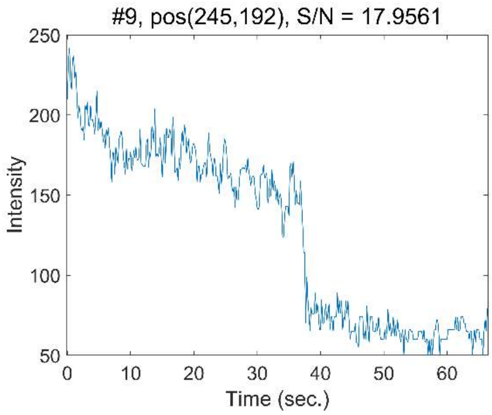
#9, pos(245,192), S/N = 17.9561
Intensity
Time (sec.)

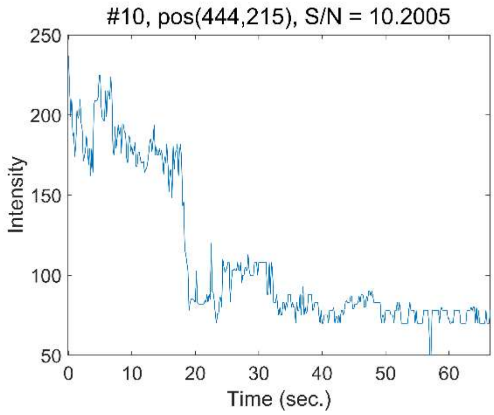
#10, pos(444,215), S/N = 10.2005
Intensity
Time (sec.)

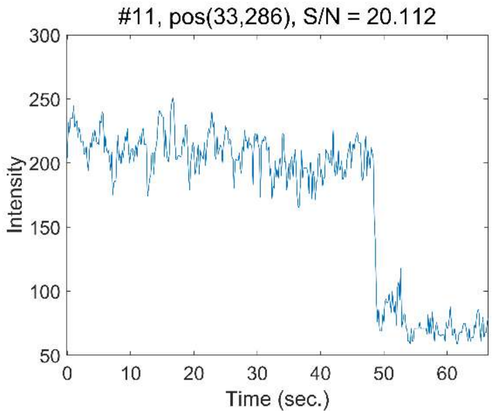
#11, pos(33,286), S/N = 20.112
Intensity
Time (sec.)

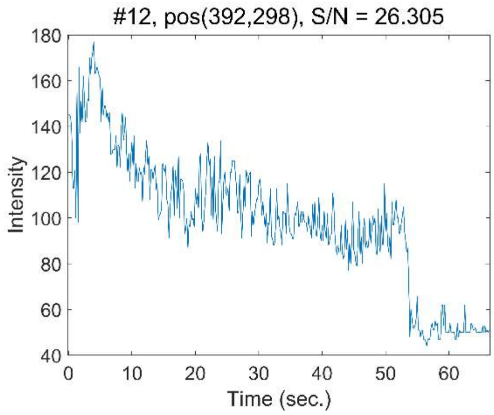
#12, pos(392,298), S/N = 26.305
Intensity
Time (sec.)

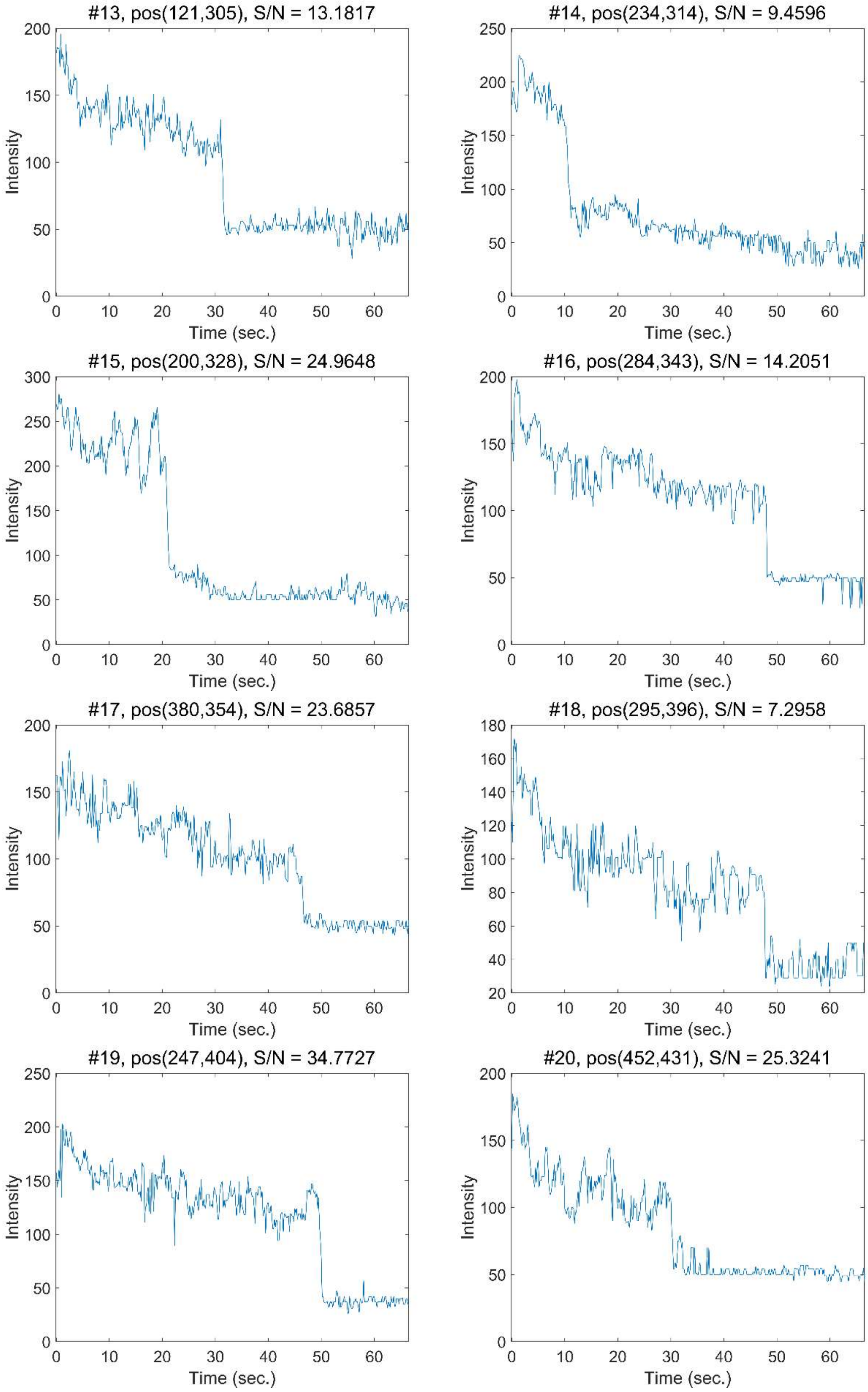
#13, pos(121,305), S/N = 13.1817
#14, pos(234,314), S/N = 9.4596
#15, pos(200,328), S/N = 24.9648
#16, pos(284,343), S/N = 14.2051
#17, pos(380,354), S/N = 23.6857
#18, pos(295,396), S/N = 7.2958
#19, pos(247,404), S/N = 34.7727
#20, pos(452,431), S/N = 25.3241
Intensity
Time (sec.)

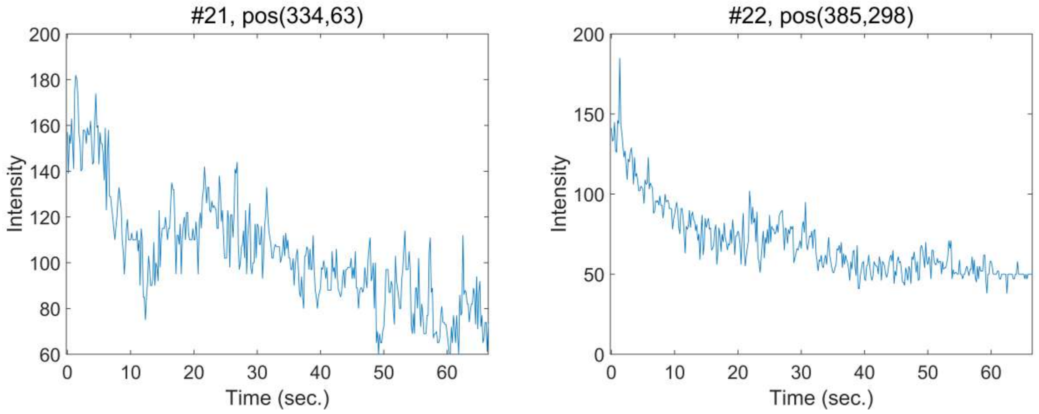

Figure S1. Randomly selected spots traces for a photobleaching experiment for Cy3. #1-#20 are single molecule traces. #21 and #22 are typical unclassified spots. The experimental condition was the same as actual sequencing process (exposure time, laser intensity, sampling frequency). The x,y coordinates (in pixels) and S/N ratio for each single spot were shown on top of each figure. The entire field-of-view was 54.6 μm x 54.6 μm. The average S/N ratio was 5.30, standard error was 0.36.

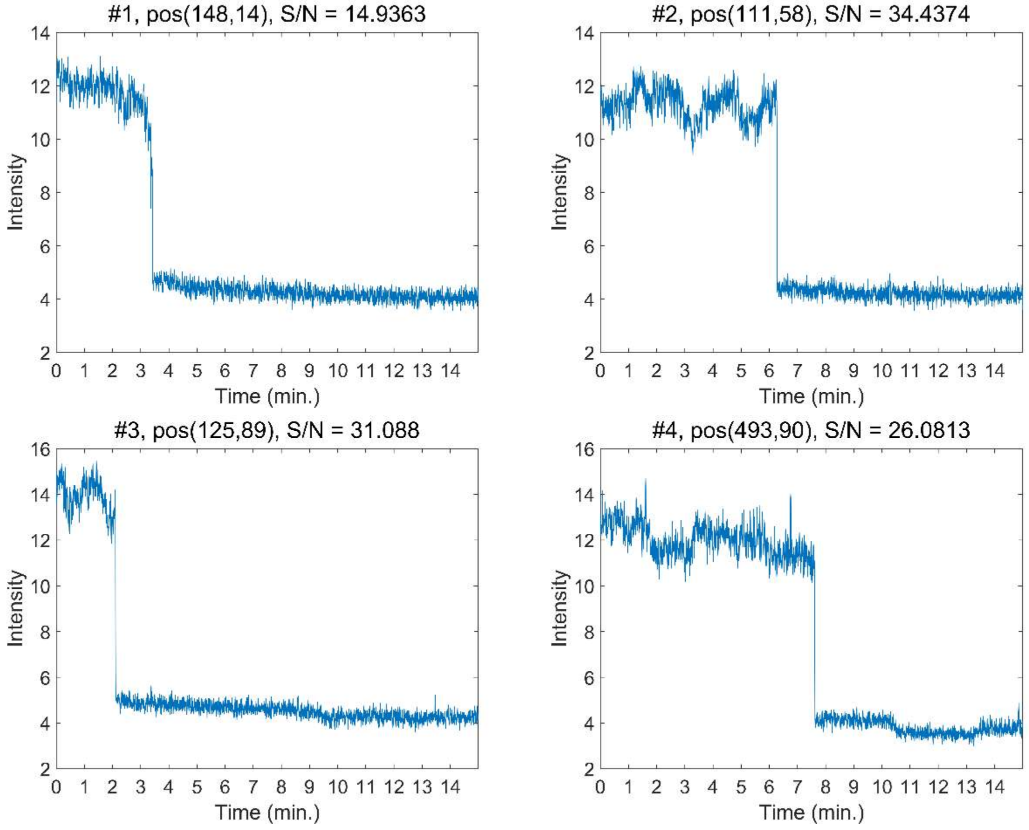

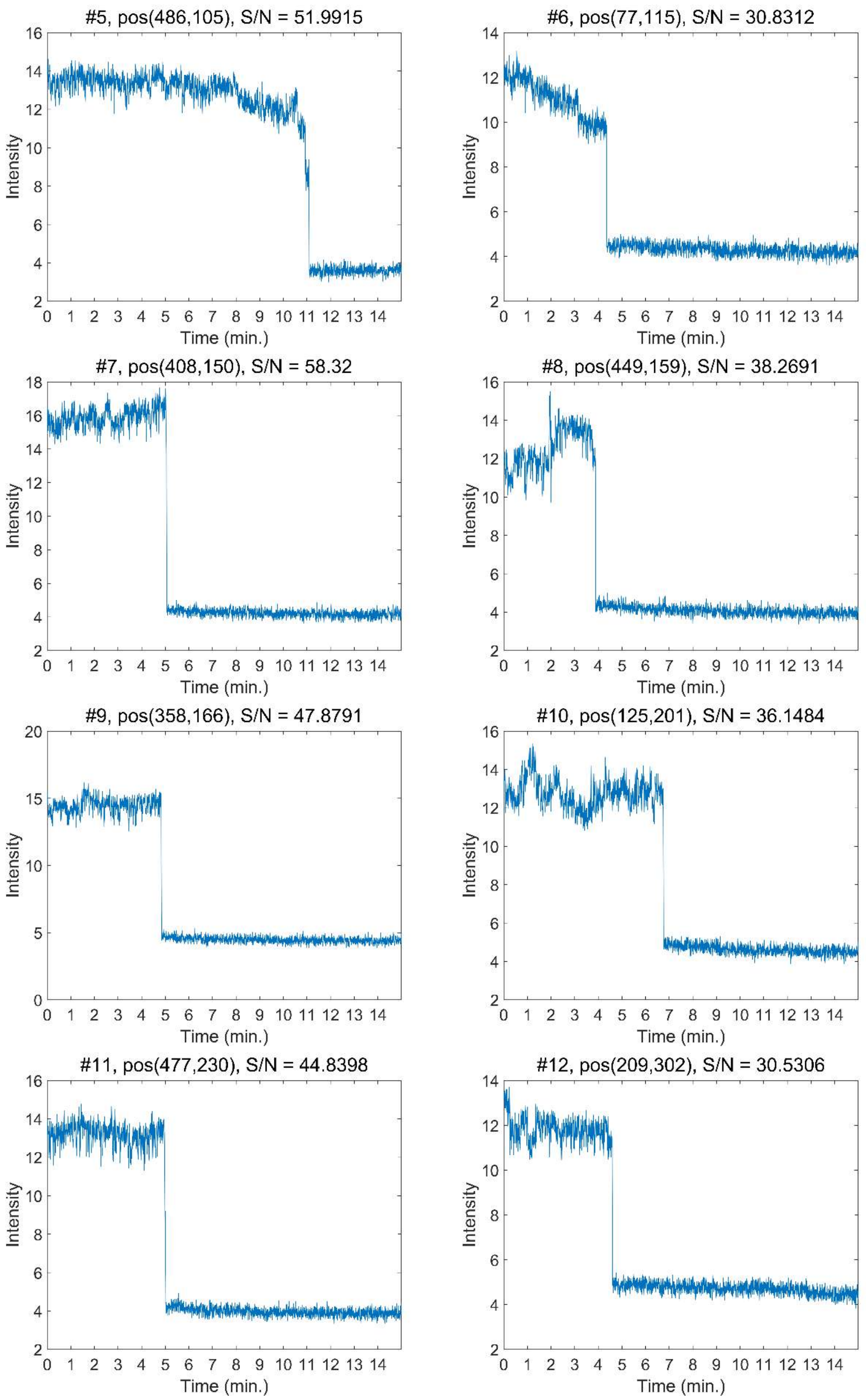
#5, pos(486,105), S/N = 51.9915
#6, pos(77,115), S/N = 30.8312
#7, pos(408,150), S/N = 58.32
#8, pos(449,159), S/N = 38.2691
#9, pos(358,166), S/N = 47.8791
#10, pos(125,201), S/N = 36.1484
#11, pos(477,230), S/N = 44.8398
#12, pos(209,302), S/N = 30.5306
Intensity
Time (min.)

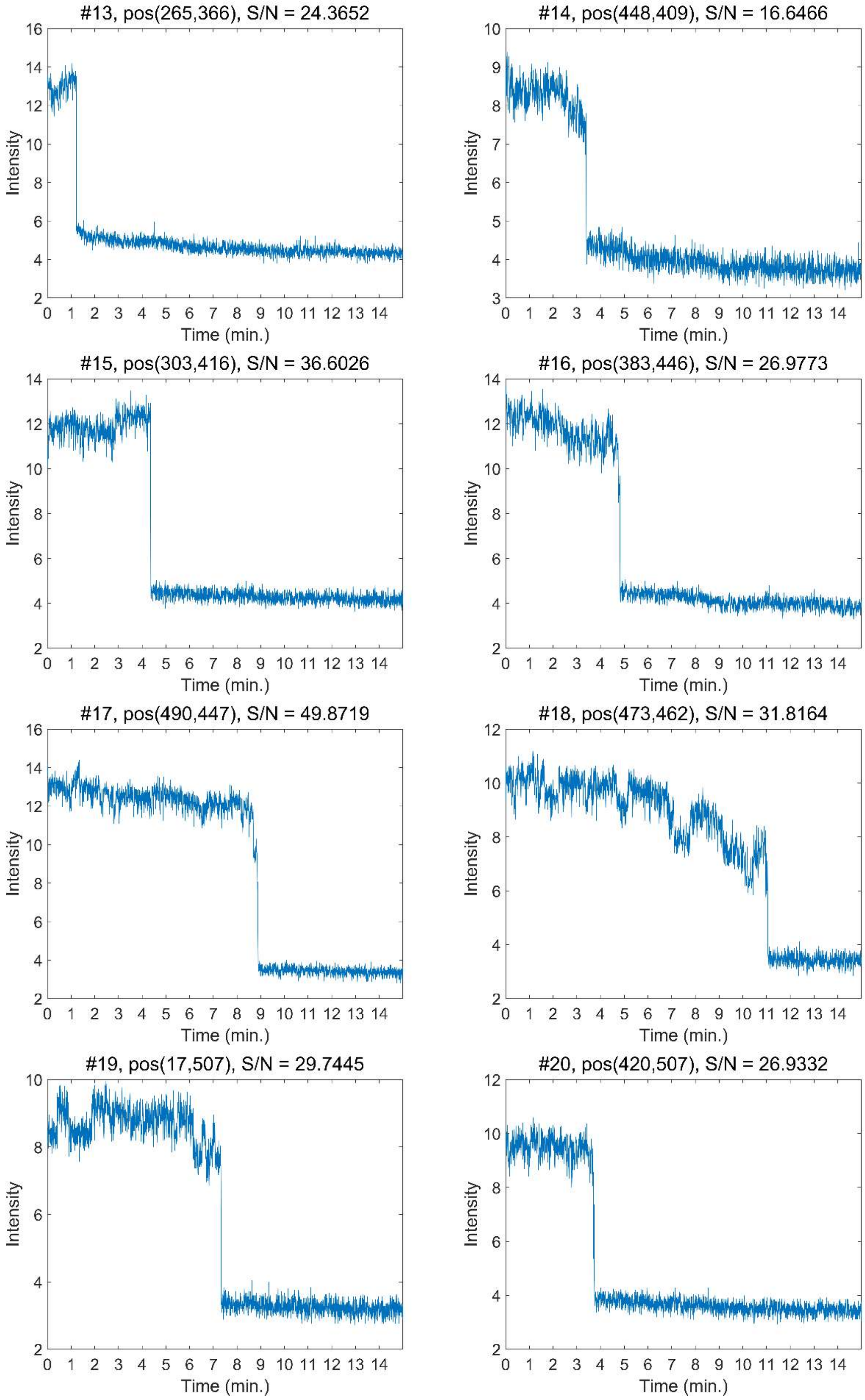
#13, pos(265,366), S/N = 24.3652
#14, pos(448,409), S/N = 16.6466
#15, pos(303,416), S/N = 36.6026
#16, pos(383,446), S/N = 26.9773
#17, pos(490,447), S/N = 49.8719
#18, pos(473,462), S/N = 31.8164
#19, pos(17,507), S/N = 29.7445
#20, pos(420,507), S/N = 26.9332
Intensity
Time (min.)

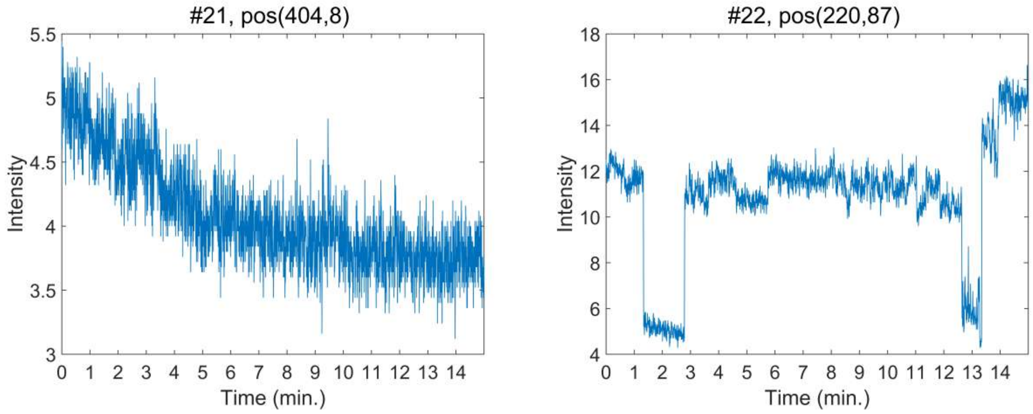

Figure S2. 20 randomly selected spot traces for a photobleaching experiment for ATTO 647N. #1-#20 are single molecule traces. #21 and #22 are typical unclassified spots. The experimental condition was the same as sequencing process (exposure time, laser intensity, sampling frequency). The x,y coordinates (in pixels) and S/N ratio for each single spot were shown on top of each figure. The entire field-of-view is 54.6 μm x 54.6 μm. The average S/N ratio was 13.52, standard error was 0.85.

Single molecule can be distinguished by tracing their dynamics of photobleaching. A single-step decreasing curve indicates single molecule, while multi-step decreasing curve indicates aggregation of dye molecules. However during the analysis of all the photobleaching curves, 26% of them cannot be classified into either single molecule or aggregated molecules. Their photobleaching curve is irregular which have been shown in Fig. S1 and S2 (#21, #22). These spots may come from the impurities on coverslip surface or reagent rather than the fluorescence dye (Cy3 or ATTO 647N)."

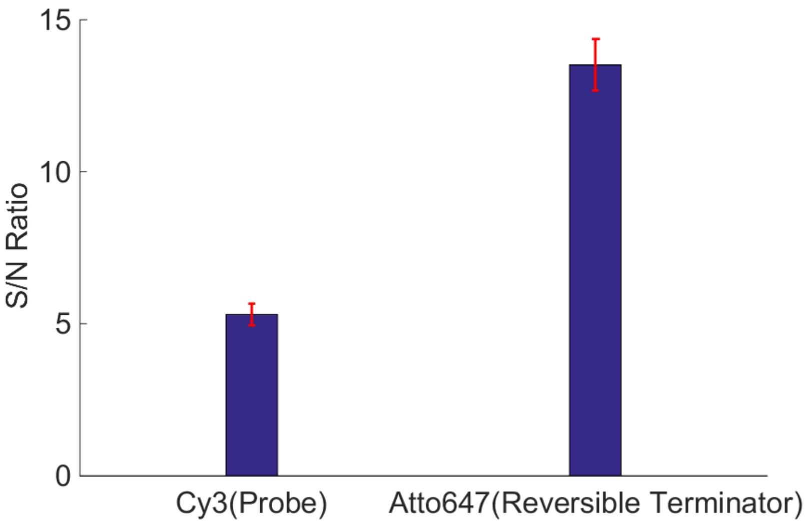

Figure S3. The average S/N ratio and standard error for both Cy3 and ATTO 647N.

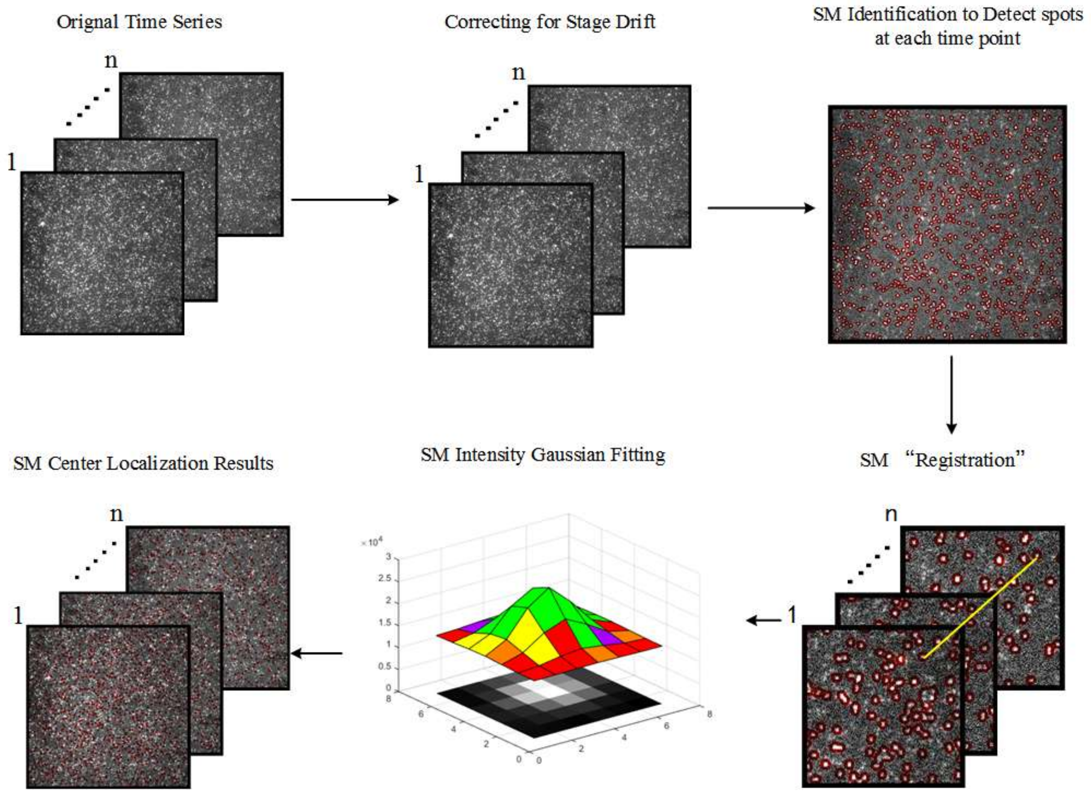

Figure S4. Schematics for imaging processing.

Increasing the photostability by imaging buffer

Crucial to the performance of single molecule experiments is the fluorophore and its lifetime. The lifetime of a fluorophore is the time it is illuminated until photobleaching occurs or until radical oxygen species damage the tethered molecules. Therefore, imaging buffers containing enzymes oxidizing substrate—so-called oxygen scavengers—are employed to create anaerobic conditions and thus increase the lifetime by preventing oxygen-based reactions. We tested the efficiency of imaging buffer by imaging capture probe with Cy3 label on the 3' prime end immobilized on the coverslips. A video was recorded to monitor the real time changes of the amount of fluorescent spots in one field of view (54.6 μm×54.6 μm, laser power 100 mW, exposure time 100 ms) in the presence of imaging buffer. A control experiment was done by using the same imaging buffer with oxygen scavenger depleted. Fig. S5 shows that imaging buffer can significantly prolong the life time of Cy3 especially when the total exposure time exceeds1min. In our experiment of sequencing, a typical exposure time for each cycle is 400 ms (100 ms×4). So 20 cycles add up to 8 s during which the loss of the spots caused by photobleaching is negligible.

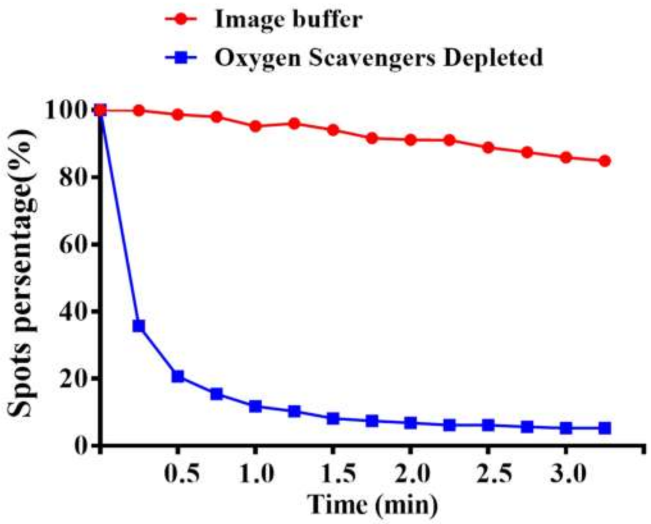

Figure S5. The efficiency of imaging buffer

Sequencing errors

The sequencing error was validated by four times of sequencing conducted under the same condition. Each single raw sequence was aligned to the reference and the number of positions disagreeing with the reference was recorded. The error rate was calculated separately for each DNA template.

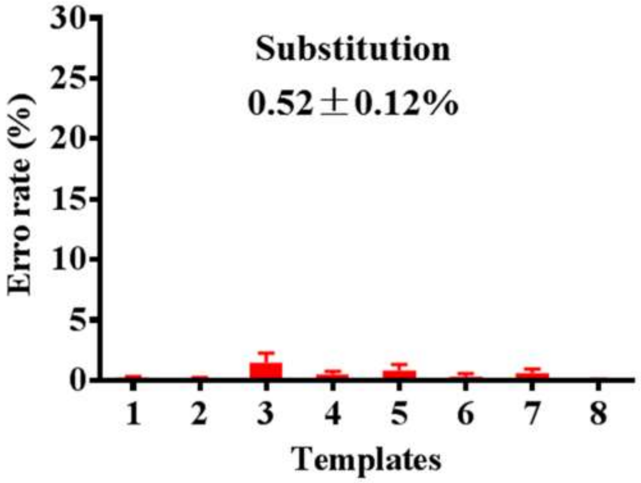

Figure S6. The average error rate in four repeated experiments. In each experiment, 8 DNA templates were mixed and sequenced. The average substitution error rate is 0.52%.
